# Sonochemical synthesized BaMoO_4_/ZnO nanocomposites as electrode materials: A comparative study on GO and GQD employed in hydrogen storage

**DOI:** 10.1016/j.ultsonch.2022.106167

**Published:** 2022-09-15

**Authors:** Fatemeh Karkeh-Abadi, Maryam Ghiyasiyan-Arani, Masoud Salavati-Niasari

**Affiliations:** Institute of Nano Science and Nano Technology, University of Kashan, P. O. Box.87317-51167, Kashan, Iran

**Keywords:** Molybdate nanostructures, Ultrasonic, Graphene, Green synthesis, Nanocomposites, Electrochemical hydrogen storage

## Abstract

Due to poor rate proficiency and electrochemical capacity of transition metal oxides, production electrode materials as operative way to develop the electrochemical performance is a crucial strategy to make sure the great electroactive sites and fast electron/ion diffusion route. In order to solve this problem, carbon-based nanocomposites as conductive substrates are applied. The nanostructured BaMoO_4_/ZnO was produced by sonochemical method in the presence of tween 20 as stabilizing agent. Effect of graphene quantum dots (GQDs) and graphene oxide (GO) for developing hydrogen capacity of BaMoO_4_/ZnO was studied by providing representative composites of BaMoO_4_/ZnO-GQDs and BaMoO_4_/ZnO-GO. For this purpose, GQDs was synthesized using green source of *Spiraea crenata* and the GO provided by commercial company*.* The structural analysis shows preparation of scales-like morphology of BaMoO_4_/ZnO without any impurities through SEM, TEM, XRD, EDS and FT-IR characterization data. Also, the specific surface area for BaMoO_4_/ZnO-GQDs (11 m^2^/g) and BaMoO_4_/ZnO-GO (124 m^2^/g) nanocomposites increased by comparing to BaMoO_4_/ZnO (9.1 m^2^/g). The resultant nanocomposites used as new active compounds for applying in hydrogen storage strategies using cyclic voltammetry and chronopotentiometry tests. Comprehensively, the hydrogen capacitance after 15 cycles was demonstrated on the nanostructured BaMoO_4_/ZnO about 129 mAhg^−1^. It demanded the maximum capacitance for BaMoO_4_/ZnO-GQDs and BaMoO_4_/ZnO-GO nanocomposites were 284 and 213 mAhg^−1^ respectively, which was higher than the initial nanostructured BaMoO_4_/ZnO. It was exposed from the carbon based structured that; the endorsed electrochemical hydrogen storage (EHS) performance is ascribed to the reaction of the redox pair of Mo^6+^ /Mo^5+^ at the active sites throughout the EHS procedure. This study delivers a novel plan and potential sorption electrode materials to progress the intrinsic action of conductive compounds.

## Highlights

• Hydrothermal synthesis of GQD with natural source of *Spiraea crenata*.
• Sonochemical synthesis of binary nanocomposites of BaMoO_4_/ZnO using tween 20.
• Design ternary nanocomposites of BaMoO_4_/ZnO-GQDs and BaMoO_4_/ZnO-GO as potential conductive electrode materials.
• Achieving higher capacity of BaMoO_4_/ZnO-GQDs (284 mAh/g) and BaMoO_4_/ZnO-GO (213 mAh/g) than BaMoO_4_/ZnO (129 mAh/g).

## 1 Introduction

The United Nations General Assembly, in its declaration of the right to development (1986), declared energy in different forms an essential need to achieve the specified targets [1]. Today, most energy needs are met through non-renewable sources [2]. The use of non-renewable resources in addition to the environmental issues due to long-term use has reached a saturation level [3]. Therefore, renewable sources for energy production at different levels are in demand. Among the renewable sources, hydrogen is one of the most attractive [4]. At the same time, this energy carrier needs efficient and secure storage to have a bright future [5]. Common hydrogen storage methods such as gas (with high-pressure), liquid (at low-temperature), and solid-state storage have been applied [6], [7]. Solid-state storage has higher security and storage capacity, repeated reversibility, and lower cost than other methods [8], [9], [10]. Carbonaceous materials, metal–organic frameworks, metal hydrides, and complex hydrides in nanomaterials-based solid-state hydrogen storage have been studied [11]. Discovery of clean and renewable energy sources has become one of the great challenges for scientific communities owing to the reduction of environmental pollution and reduction reliance on oil and major reduction in air pollution [12]. Hydrogen is recognized as one of the clean energy sources which does not produce any pollution and greenhouse gases [13], [14]. Hydrogen is propounding as one of the most promising energy carriers (not an energy source) in the future due to its safety, eco-friendly, abundance, and high energy density. Hydrogen storage takes place in various systems, including porous and layered materials (Silica and alumina materials, Carbon materials, porous polymers, Metal-organic framework materials, Pillared clay, metal oxide and etc.) [15], [16], [17], [18]. Hydrogen storage categorization on the basis of the procedure is sorted into two avenues of (i) Adsorption based storage and (ii) Absorption based storage. The mechanism in adsorption-based systems can describe via electrochemical reactions. At the working electrode, water itself is the source of protons in the electrolyte solution which leads to OH^–^ as following electron reaction (Eq. (1)) and the nascent hydrogen is adsorbed on the surface of adsorbent (A) in the Eq. (2) or recombined to H_2_ molecules as Eqs. ((3)–(5)).

(1) $$H_{2}O+e^{-}\to H+OH^{-}$$

(2) $$A+H\to AH_{\mathrm{ads}}$$

(3) $$AH_{\mathrm{ads}}+H_{2}O+e-\to H_{2}+OH^{-}+A$$

(4) $$2H\to H_{2}$$

(5) $$AH_{\mathrm{ads}}+\;AH_{\mathrm{ads}}\to \;H_{2}+2A$$

The mentioned equation of (1), (3), (4) are Volmer, Heyrovsky, and Tafel reactions represented for different stages in the electrochemical process [19], [20], [21]. If the energy released in the Tafel or Heyrovsky reactions is less than the energy generated in the hydrogen adsorption reaction, and the activation barrier for one of these reactions is low enough, H_ads_ recombination occurs, and molecular hydrogen develops electrochemically. If not, the adsorbed hydrogen might migrate further into the adsorbent, occupying sites with higher adsorption energy [22].

In the last decades, carbon materials consisting of 0-dimensional, 1-dimensional, 2-dimensional, 3-dimensional structures (i.e., CQDs and GQDs, carbon nanotubes, graphene, graphite, and graphite oxide (GO)) have been applied to vast research areas specially hydrogen storage area because of extremely useful properties [23], [24], [25], [26]. Graphite oxide is a multi-layered carbon structure obtained by oxidation of graphite with acid [27]. Graphite oxide has unique properties such as controllable surface structure, different functional groups, change in the distance of graphite plates ∼ 6–7 Å due to reaction with acid, and carbon atoms with hybridization sp^2^ and sp^3^ [28], [29], [30]. Wang et al. found that palladium-doped graphite oxide performed in hydrogen storage is better than graphite oxide [31]. With reducing size in all three dimensions, unique structures were formed with a platform consisting of one or more graphene layers and a graphite-like crystal structure which called graphene quantum dots (GQDs) [32], [33], [34], [35]. Graphene quantum dots with oxygenated functional groups, edge effects, and high surface area have particular appeal in electrochemical applications [36], [37], [38]. GQDs have been widely used in various fields such as lithium batteries, supercapacitors, photocatalysts, and antibacterial [39], [40], [41], [42]. Recently, researchers studied N, S-GQDs in hydrogen storage. The results exhibited that *N*-GQDs and S-GQDs improved hydrogen storage performance [43].

An important series of inorganic materials are binary metal oxides molybdates (AMoO_4_, A: Mg, Ba, Sr, Ni, Cu, Co, Zn, and so on). Molybdenum can exist in four oxidation states (Mo^0^, Mo^+2^, Mo^+4^, Mo^+6^) and forms various stable oxides [44]. Among these compounds, barium molybdate is synthesized by various methods such as solid-state, co-precipitated, and hydrothermal [45], [46], [47]. BaMoO_4_ structure is very sensitive and depends on the synthesis conditions. The luminescence, dielectric, and electrocatalytic properties of barium molybdate have been evaluated [46], [48], [49]. To the best of our knowledge, research has not been done on barium molybdate and its composites for hydrogen storage. However, there are other oxide materials that have attracted much attention in the electrochemical storage of hydrogen due to structural properties, and ZnO is one of them. The surface properties, wide bandgap, and high exciton bond energy cause attractive electrochemical hydrogen energy storage characteristics in ZnO [50], [51]. The potential of ZnO in different conditions (e.g., ZnO and Mg-doped ZnO [52], change in weight percentage of Mg-doped ZnO nanowires [53], ZnO in various morphologies [54], ZnO@f-MWCNTs [55], ZnO-MWCNTs [56], Al and Mg-doped ZnO nanofibers [57], LiBH_4_@x ZnO/ZnCo_2_O_4_ [58], ZnO-HMD@ZnO-Fe/Cu core–shell [51], Mo/DAN/ZnO [59]) have been tested in hydrogen storage. Inspired by these investigations, can be designed new structures and evaluated in hydrogen energy storage.

In the current research, the BaMoO_4_/ZnO nanocomposite synthesized by sonochemical method in the short time and in the presence of tween 20 capping agent. Then, the introduced nanocomposites were examined as a novel electrode for hydrogen energy storage. The effect of zero-dimensional and two-dimensional carbon compounds in hydrogen energy storage with the motivation to investigate and target future research was evaluated by introducing two new three-component nanocomposites (BaMoO_4_/ZnO-GQDs and BaMoO_4_/ZnO-GO). The resultant carbon-based nanocomposites compared with BaMoO_4_/ZnO for hydrogen capacity using electrochemical methods. So, the structural and physical properties were studied for better characterization.

In the current study, the nanocomposites of BaMoO_4_/ZnO-GQDs and BaMoO_4_/ZnO-GO designed for electrochemical hydrogen storage application. The principal aims are perceived following:

1) The sonochemical synthesis has been applied to formation of nanostructured BaMoO_4_/ZnO in the presence of tween 20.
2) For the first time, the graphene quantum dot was prepared from new natural source of *Spiraea crenata* through hydrothermal technique.
3) The designed nanocomposites utilized as electrode materials in the chronopotentiometry test for comparing the electrochemical hydrogen storage efficiency of nanocomposites which provided by GQD or GO for comparing studies.
4) The designed nanocomposites were utilized for hydrogen storage, for initial time to obtain the purpose of storing hydrogen. We have applied these nanocomposites as new and effective agent for potential active materials.

## 2 Experimental procedure

### 2.1 Materials

The graphene quantum dot was prepared using *Spiraea crenata* collected from Kaleybar-Azerbaijan-IRAN. BaMoO_4_/ZnO was fabricated using barium nitrate (Merck), zinc nitrate hexahydrate (Aldrich), sodium molybdate dihydrate (Merck), and tween 20 (Merck). Eventually, potassium hydroxide and ethanol (Merck) were used for the electrochemical hydrogen storage studies. The chemical precursors and starting materials utilized in synthesis process of samples were purchased from a Merck or Aldrich company and applied as arrived without further purification.

### 2.2 Fabrication of graphene quantum dots

The GQDs were synthesized *via* the hydrothermal procedure using the green source of *Spiraea crenata*. Collected *Spiraea crenata* were washed with DI water for several times and dried in sunlight for 14 days. Subsequently, *Spiraea crenata* was powdered using a home mill. 5 g of this powder was dispersed in 50 ml of ultrapure water and stirred for 24 h at room temperature. The resulting uniform suspension was transferred to a Teflon-lined stainless autoclave and kept in an oven at 180 °C for 24 h. Eventually, the obtained brown colored solution was centrifuged five times at 15,000 rpm for 60 min, and the final product was dried in a vacuum freeze-dryer for 24 h.

### 2.3 Synthesis of nanostructured BaMoO_4_/ZnO

The sonochemical technique was applied for the preparation of nanostructured BaMoO_4_/ZnO. For this purpose, 3 g barium nitrate, 2.78 g sodium molybdate dihydrate, 2.17 g zinc nitrate hexahydrate, and 2.5 cc tween 20 (polysorbate) were dissolved in DI water/ethanol (in a ratio 2:1 v/v) and well mixed up. The tween 20 plays the role of capping agent in the reaction process. The pH of mixture was adjusted using ammonia about 8–9. Next, the resulting mixture was placed in an ultrasonic device (Hielscher, UP400S, 400 W, 24 kHz) for 10 min. The observed white colored suspension confirms the formation of nanostructured BaMoO_4_/ZnO. After separation using a high-speed centrifuge, the resulting nanocomposite was washed various times with ultra-pure water and dried in an oven at 60 °C for 12 h. Finally, the samples were calcined for 2 h at 400 °C to remove organic materials and form a crystalline phase.

### 2.4 Synthesis of BaMoO_4_/Zn-GQDs nanocomposites

The as-synthesized BaMoO_4_/ZnO nano powders and GQDs were dispersed in ethanol by ultrasonic bath in a fixed weight ratio of 1:1. This mixture was irradiated under microwave power of 500 W until the color of the mixture became uniformly yellowish brown. Finally, the obtained nanocomposites were separated by filtration and dried at ambient temperature.

### 2.5 Synthesis of BaMoO_4_/ZnO-GO nanocomposites

To preparation of BaMoO_4_/ZnO-GO nanocomposites, the graphite oxide was initially fabricated with a modified Hummers method [60], [61]. In the second step, 1 g of graphite oxide and 1 g of BaMoO_4_/ZnO were dispersed in ethanol and stirred for 2 h at 60 °C. The above homogeneous suspension was placed under microwave radiation for 10 min at a power of 750 W (LG, MH8265DIS(. After collecting, the blackish gray product was dried in an oven at 70 °C.

### 2.6 Electrochemical hydrogen storage

All examinations counting for the measured provided materials was probed to comparison of models in terms of electrochemical activity for exploiting in energy storage demand. The electrochemical cell collected by means of potassium hydroxide (2 M) as electrolyte and three-electrodes of copper based working electrode, Pt counter electrode and Ag/AgCl reference electrode, compatibly. The constant current was emploed in the gathered cell between the providing working electrode and counter electrodes and the potential change between the providing working electrode and the reference electrode was protected. The working electrode provided by coating the suspended active materials on the copper substrate with surface area of 1 cm^2^.

### 2.7 Techniques of characterizations

Fourier transform infrared (FTIR) spectroscopy was recorded to recognize the chemical construction of nanostructures prepared with instruments (Perkin-Elmer 400 spectrometer, UK). The study of the structural properties of the prepared samples was performed by an X-ray diffractometer (XRD, X’Pert PRO MPD, Netherlands) *via* radiation Ni-filtered Cu Kα (λ = 1.5406 Å) in the 2θ = 5-80°. The BET (Brunauer–Emmet–Teller) analysis was performed to evaluate the specific surface area and porosity of nanocomposites fabricated by device (Belsorp mini II, Bel, Japan) adsorption and desorption of N_2_ at −196 °C. Pore size distribution was measured using the N_2_ desorption isotherm and method Barrett, Joyner, and Halenda (BJH). The field emission scanning electron microscope machine (Sigma, Zeiss, Germany), X-ray energy diffraction spectroscopy and elemental mapping (Oxford Instruments, UK) evaluated the surface morphological, microstructure and distribution elements. TEM images were captured on a Philips EM208 transmission electron microscope with an accelerating voltage of 200 kV. The electrochemical measurements were performed by the potentiostat/galvanostat (SAMA 500, electroanalyzer system, I.R. Iran).

## 3 Results and discussion

### 3.1 Fourier transform infrared (FTIR) analysis

**Fig. 1:**
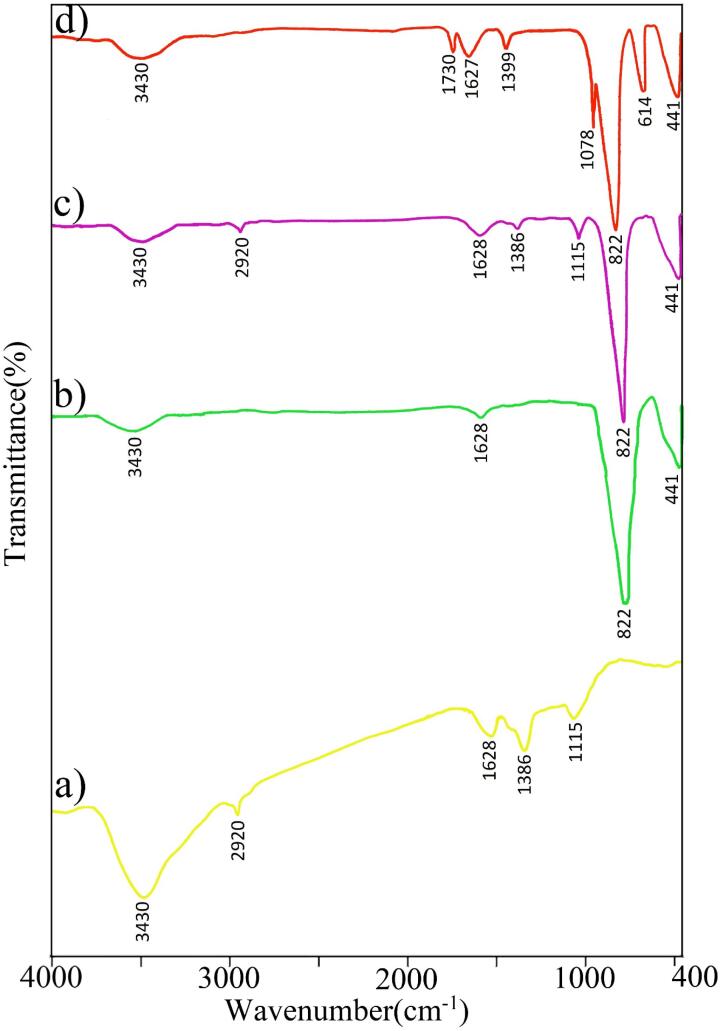
FT-IR spectra for (a) GQDs, (b) BaMoO_4_/ZnO, (c) BaMoO_4_/ZnO-GQDs and (d) BaMoO_4_/ZnO-GO.

The vibrations of the prepared nanostructures were investigated by the important technique of Fourier Transform IR spectroscopy, and the results are shown in Fig. 1. For GQDs, the broad peak at 3430 cm^−1^ corresponded to the stretching vibration of O—H [62]. The band at 2920 cm^−1^ was ascribed to the stretching vibrations of the -C—H [63]. Absorption peaks in 1628 cm^−1^ are related to aromatic ring stretching C=C [64], [65]. The three bands at 1386 and 1115 cm^−1^ could be attributed to the C=O and C-OH groups, respectively [66], [67], [68], [69]. For BaMoO_4_/ZnO nanocomposites, the peaks at 3430 and 1628 cm^−1^ indicate the symmetric-antisymmetric stretching vibrations and bending of the water molecules adsorbed on the material surface, which is derived from moisture [70], [71]. The peak at 822 cm^−1^ represents the stretching vibrations mode Mo-O [49]. The peak at 441 cm^−1^ has been assigned to the metal–oxygen vibrations (M−O, M: Zn, Ba) [72]. In addition, it can indicate a weak bending vibration Mo-O [49]. The FT-IR spectrum for BaMoO_4_/ZnO-GQDs nanocomposite shows all BaMoO_4_/ZnO and GQDs absorption peaks, which confirm the successful synthesis. In spectrum BaMoO_4_/ZnO-GO, a similar trend was observed for bands BaMoO_4_/ZnO. Furthermore, the bonds for C=O carboxyl or carbonyl groups, C=C stretching vibration, O—H stretching vibration, C—O—C, -C—H bending vibration were located at wavenumbers of 1730, 1627, 1399, 1078, 614 cm^−1^, which demonstrates the successful introduction of graphite oxide [73], [74], [75].

### 3.2 X-ray diffraction (XRD) analysis

**Fig. 2:**
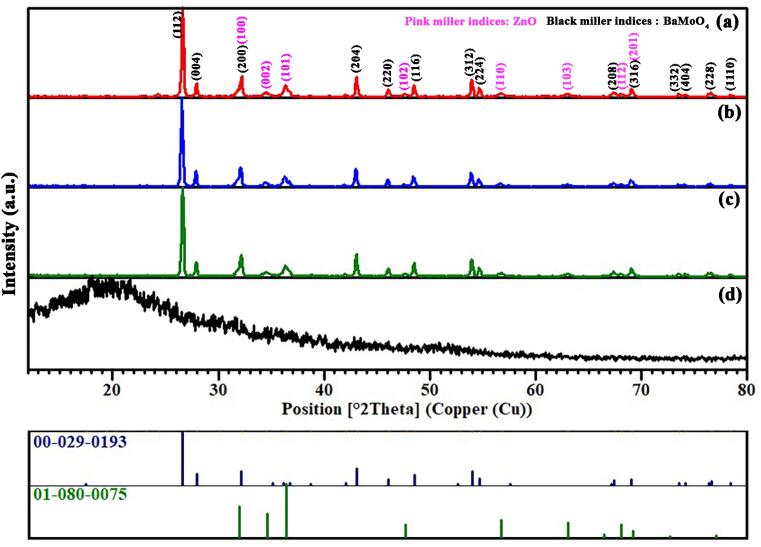
XRD pattern for resultant samples (a) BaMoO_4_/ZnO, (b) BaMoO_4_/ZnO-GQDs, (c) BaMoO_4_/ZnO-GO and (d) GQDs.

The X-ray diffraction patterns of the nanostructured BaMoO_4_/ZnO, BaMoO_4_/ZnO-GQDs, BaMoO_4_/ZnO-GO and GQDs are presented in Fig. 2 (a-d). The GQDs exhibited an extensively wide peak at the center of 20°originating from the amorphous structure and graphitic carbon texture [76]. For BaMoO_4_/ZnO nanocomposite, the diffractogram can be indexed by the crystal structures tetragonal phase of BaMoO_4_ (JCPDS card no: 0–029-0193) with space group *I*41/*a* and the hexagonal phase of ZnO (JCPDS card no:01–080-0075) with space group *P*63*mc* without any impurities in the crystal system. The average crystal domain size (Dc) in nm of BaMoO_4_/ZnO was calculated by using the Debye-Scherrer equation (Eq. (6)) [77]:

(6) Dc=Kl/(bCosq)

where K, λ, β, and θ are the shape factor that generally takes a value of 0.9, X-ray wavelength (0.154 nm), peak width at half maximum height (FWHM), and diffraction angle, respectively. The average crystal domain size has obtained 34 nm for BaMoO_4_/ZnO. XRD results confirm the successful fabrication of crystalline phases of the introduced carbon based-nanocomposites.

### 3.3 Shape and surface classification

**Fig. 3:**
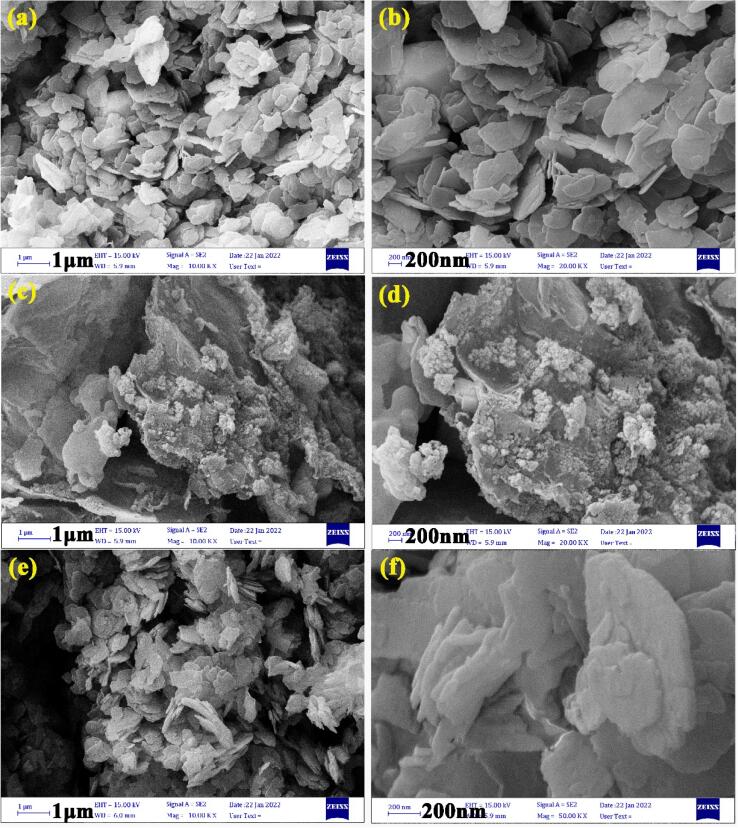
Morphological study of resultant electrode materials using Field Emission Scanning Electron Microscope (a, b) BaMoO_4_/ZnO, (c, d) BaMoO_4_/ZnO-GO and (e, f) BaMoO_4_/ZnO-GQDs.

**Fig. 4:**
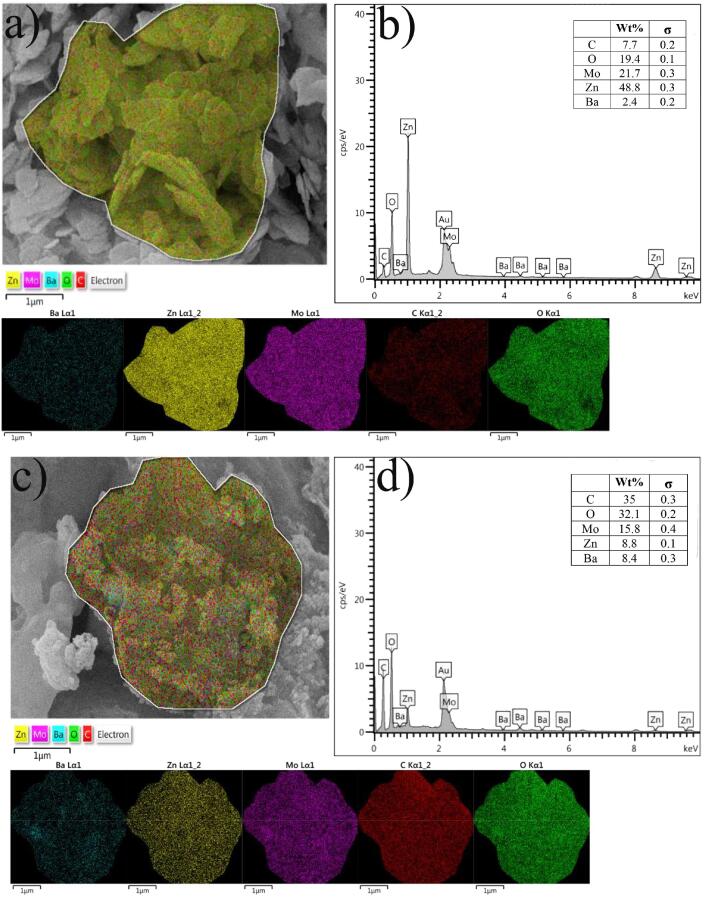
Elemental mapping and EDS analysis for (a, b) BaMoO_4_/ZnO-GQDs and (c, d) BaMoO_4_/ZnO-GO.

Fig. 3 depicts the surface morphology of provided BaMoO_4_/ZnO, BaMoO_4_/ZnO-GQDs, and BaMoO_4_/ZnO-GO nanocomposites at two scales (1 µm and 200 nm). FE-SEM images of BaMoO_4_/ZnO (Fig. 3a, b) show scales-like morphology with nanometer thickness. The FESEM image of BaMoO_4_/ZnO-GO (Fig. 3b, c) shows that sheet-like graphite oxide structure has not deformed during the manufacturing process. The rough surface of graphite oxide are suitable for electrochemical hydrogen storage due to the presence of BaMoO_4_/ZnO with the characteristics of the larger specific surface and active sites. According to the Fig. 3(e, f), the observations confirm that GQDs are covered on the surface of scales-like BaMoO_4_/ZnO without any detectable deformation in morphology. The EDS and elemental mapping studies evaluated the microstructure in samples BaMoO_4_/ZnO-GQDs (Fig. 4(a, b)) and BaMoO_4_/ZnO-GO (Fig. 4(c, d)). The samples were coated with gold to create electrical conductivity before preparing the images. The elements of Ba, Mo, Zn, O, and C are related to BaMoO_4_/ZnO, GQDs, and GO chemical composition. The distribution of the above elements at the sample surface is confirmed by elemental map analysis.

**Fig. 5:**
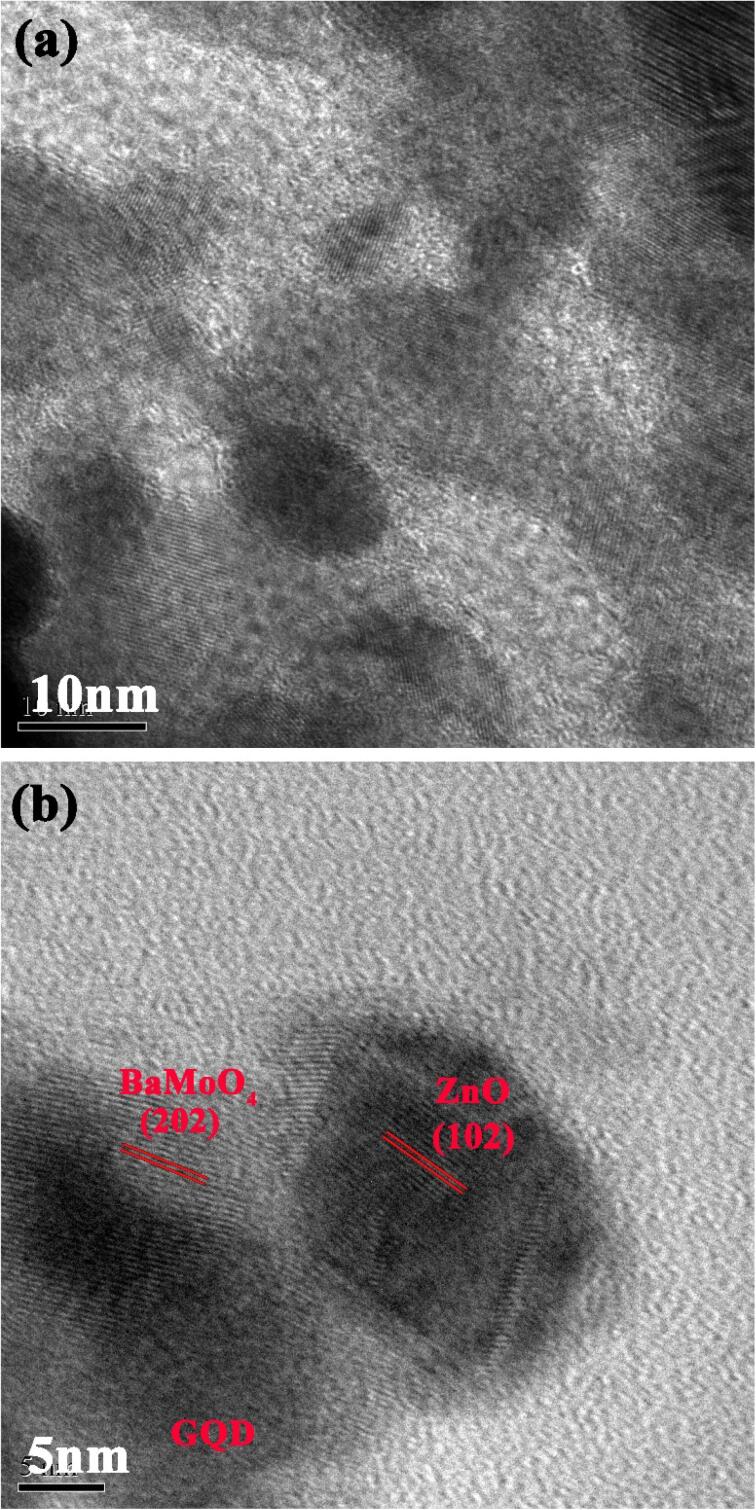
Morphological and crystal structure study of resultant BaMoO_4_/ZnO-GQDs sample using Transmission electron microscopy.

Additionally, the morphology and phase structure of BaMoO_4_/ZnO-GQDs nanocomposites were inquired by TEM and HR-TEM images (Fig. 5(a, b)). The obtained amorphous phase related to the graphene quantum dots and the both crystal phase of BaMoO_4_ and ZnO specified in the Fig. 5b with lattice fringe spacing. The space fringes of 0.191 and 0.256 nm correlated to lattice spacing facet of (2 0 2) BaMoO_4_ and (1 0 2) ZnO respectively.

Nanosized BaMoO_4_/ZnO nanocomposites attained in preparation process seem possible owed to the creation of inspiring emulsion droplets persuaded by sonochemical irradiations. Under sonochemical irradiations, aqueous phase comprising precursor salts is spread in tween 20 as oil phase to cration of small droplets. A shock wave makes owed to implosive collapse of bubbles which distributes into the surrounding liquid. Shock irradiations can hasten spread nanoparticles in the liquid. Interparticle collisions can reach velocities of hundreds of meters per second and particle fragmentation is perceived. Sonication power can alter the structural properties of products such as size and homogeneity [17]. So, the formation mechanism occurred as follows equations (7–13):

(7) $$NH_{3}\left(\mathrm{aq}\right)\;+\;H_{2}O\;\left(l\right)+\text{ultrasonic irradiation}\to \;NH_{4}^{+}\left(\mathrm{aq}\right)+\;OH^{-}-\left(\mathrm{aq}\right)$$

(8) $$\mathrm{Ba}{\left(NO_{3}\right)}_{2}\left(s\right)\;↔\;Ba^{2+}\left(\mathrm{aq}\right)+\;2NO_{3}^{-}\left(\mathrm{aq}\right)$$

(9) $$\mathrm{Zn}{\left(NO_{3}\right)}_{2}.6H_{2}O\;\left(s\right)\;↔\;Zn^{2+}\left(\mathrm{aq}\right)+\;2NO_{3}^{-}\left(\mathrm{aq}\right)\;+\;6H_{2}O\;\left(l\right)$$

(10) $$\mathrm{NaMo}O_{4}.2H_{2}O\;\left(s\right)\;↔\;Na^{2+}\left(\mathrm{aq}\right)\;+\;MoO_{4}^{2-}\left(\mathrm{aq}\right)\;+\;2H_{2}O\;\left(l\right)$$

(11) $$\mathrm{Mo}O_{4}^{2-}\left(\mathrm{aq}\right)\;+\;Ba^{2+}\left(\mathrm{aq}\right)\;+ultrasonic\;irradiation\;\to \;BaMoO_{4}\left(s\right)$$

(12) $$Zn^{2+}\left(\mathrm{aq}\right)\;+\;OH^{-}\left(\mathrm{aq}\right)\;↔\;Zn{\left(\mathrm{OH}\right)}_{2}\left(\mathrm{aq}\right)$$

(13) $$\mathrm{Zn}{\left(\mathrm{OH}\right)}_{2}\left(\mathrm{aq}\right)\;+\;\text{ultrasonic}\;\text{irradiation}\to \;ZnO\;\left(s\right)\;+\;H_{2}O\;\left(l\right)$$

The green synthesis of graphene dots mainly focuses on the selection of green raw materials, i.e., biomass materials, and the development of sustainable synthesis techniques. In comparison with toxic organic molecules such as aromatic hydrocarbon compounds, biomass is safer, cheaper and easier to obtain. Currently, biomass is mainly divided into three types: organism, waste materials and protein products. The utilization of renewable and waste materials in the synthesis of carbon dots is consistent with the idea of a sustainable development strategy. In addition to biomass synthesis, the sustainable synthesis technique is also an important part of the green synthesis of carbon dots, including base catalysis, self-exothermic synthesis and reduction methods [78]. In this work, the hydrothermal method was applied for synthesis of graphene quantum dot with source of *Spiraea crenata.* The synthesis was occurred in the electric oven using autoclave. There are several hydrothermal syntheses of carbon dots or graphene dots which synthesized by green source in the same conditions which listed in the Table 1.

**Table 1:** Summarizing the carbon dots materials prepared by using different green source.

Material	Green source	Synthesis Method	Synthesis Condition	Ref.
*GQDs	Corn Powder as carbon source	Hydrothermal	(200 °C, 10 h)	[33]
GQDs	Coffee grounds as carbon source	Hydrothermal	(150–200 °C, 6–10 h)	[84]
GQDs	Grass waste as carbon source	Hydrothermal	(200 °C, 8 h)	[85]
GQDs	Brewer’s spent grains as carbon source	Hydrothermal carbonization	(100 °C, 24 h)	[86]
N, S-GQDs	Natural honey as carbon source, garlic as sulfur source, and ammonia as a nitrogen source.	Hydrothermal	(200 °C, 6 h)	[87]
GQDs	Lignin	Hydrothermal	(180 °C, 12 h)	[88]
**CDs	Saffron	Hydrothermal	(200 °C, 14 h)	[89]
CDs	Solanum tuberosum (Potato)	Hydrothermal	(170 °C, 12 h)	[90]
CDs	prawn shells	Hydrothermal	(200 °C, 8 h)	[91]
***CQDs	Turmeric	Hydrothermal	180–200 °C for 10–48 h	[92]
CDs	lemon juice	Hydrothermal	180 °C for 24 h	[93]

*GQDs = Graphene quantum dots.

**CDs = Carbon dots.

***Carbon quantum dots.

**Fig. 6:**
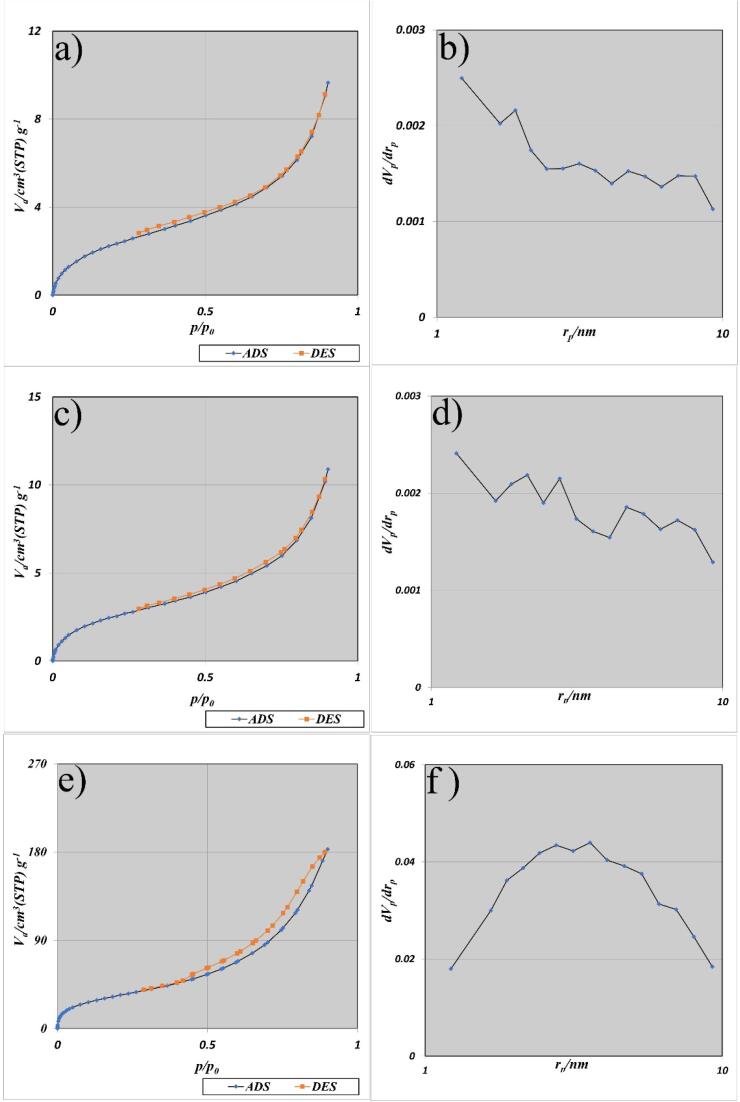
BET-BJH results for (a,b**)** BaMoO_4_/ZnO, (c, d) BaMoO_4_/ZnO-GQDs and (e, f) BaMoO_4_/ZnO-GO.

The BET-BJH analysis is a standard method for determining surface and porosity characteristics using nitrogen gas sorption. The sorption isotherms, BJH curves, and summarized characteristics of the prepared BaMoO_4_/ZnO, BaMoO_4_/ZnO-GQDs, and BaMoO_4_/ZnO-GO nanocomposites are pinpoints in Fig. 6 and Table 2. As can be seen, all three samples show adsorption isotherms of type IV and hysteresis of type H3 which confirm the mesoporous solid structures and slitted pores with non-uniform size distribution [79]. In comparison among BaMoO_4_/ZnO, BaMoO_4_/ZnO-GO and BaMoO_4_/ZnO-GQDs, the large specific surface area related to the BaMoO_4_/ZnO-GO nanocomposites because of the large specific surface area of graphite oxide[80]. The large specific surface area of the BaMoO_4_/ZnO-GO nanocomposites provides more active sites for electrochemical hydrogen storage, which causes the process of electrochemical hydrogen storage to be more efficient. BJH plots show the pore size distribution for BaMoO_4_/ZnO, BaMoO_4_/ZnO-GQDs, and BaMoO_4_/ZnO-GO samples; are matched with the BET technique data.

**Table 2:** Surface properties of the nanostructures.

Sample	BET surface area (m^2^/g)	Mean pore diameter (nm)	Total pore volume (mL/g)
BaMoO_4_/ZnO BaMoO_4_/ZnO-GQDs BaMoO_4_/ZnO-GO	9.1 11 124	6.568 6.8 9.14	0.015 0.017 0.280

### 3.4 Electrochemical studies

Activity of resultant electrode materials based on BaMoO_4_/ZnO-GO and BaMoO_4_/ZnO-GQDs were compared with BaMoO_4_/ZnO in terms of electrochemical behaviors using cyclic voltammetry (CV) and charge–discharge chronopotentiometry (CP) techniques. The conventional CV and CP set was assembled in the 2 M potassium hydroxide electrolyte and three-electrodes of working, counter (Pt) and reference (Ag/AgCl). The CV test was conducted with scan rate of 0.1 V s^−1^. During charge–discharge CP test, the constant current of 1 mA applied between the working and counter electrodes and the potential of working electrode was recorded versus reference electrode.

**Fig. 7:**
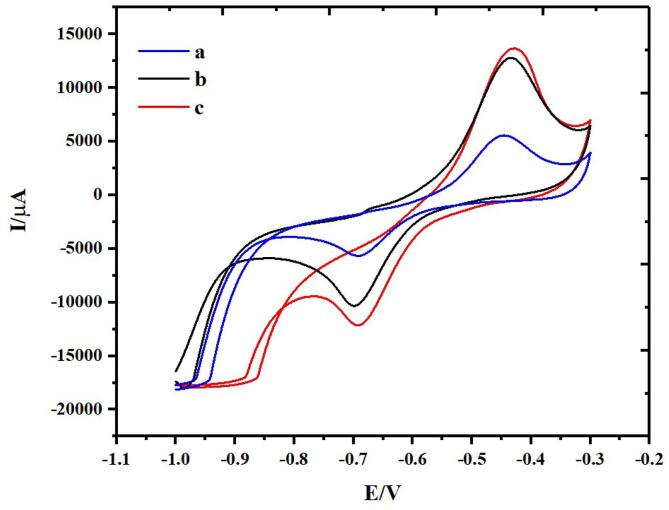
Cyclic voltammograms for resultant samples (a) BaMoO_4_/ZnO, (b) BaMoO_4_/ZnO-GO and (c) BaMoO_4_/ZnO-GQDs.

According to the obtained cyclic voltammograms in Fig. 7, all provided electrode materials for hydrogen storage verify reversible curve within the potential range − 1.0 to − 0.3 V. Table 3 defines the anodic and cathodic feedback data. Presence of Graphene oxide or GQDs alter the electrochemical feedbacks of BaMoO_4_/ZnO due to suitable conductivity and structural properties of grapheme materials. The attained feedback for BaMoO_4_/ZnO-GO and BaMoO_4_/ZnO-GQDs is higher than BaMoO_4_/ZnO. The anodic current for BaMoO_4_/ZnO, BaMoO_4_/ZnO-GO and BaMoO_4_/ZnO-GQDs are 5542, 12,764 and 13,638 µA respectively. The attained anodic and cathodic feedbacks are potential peaks for active materials which noticeably reflected recommendable electrochemical characteristic for hydrogen storage. Besides, cathodic feedback is correlated to adsorption of hydrogen on nanostructures and anodic feedback can be ascribed to reversible adsorption/desorption process happening on the candidate electrodes.

**Table 3:** Electrode response for resultant samples.

Electrode No.	Electrode Feedback			
	IPa/µA	EPa/V	IPc/µA	EPc/V
BaMoO4/ZnO	5542.8	−0.448	−5648.8	−0.688
BaMoO4/ZnO-GO	12,764	−0.437	−10307	−0.696
BaMoO4/ZnO-GQDs	13,638	−0.431	−12143	−0.692

**Fig. 8:**
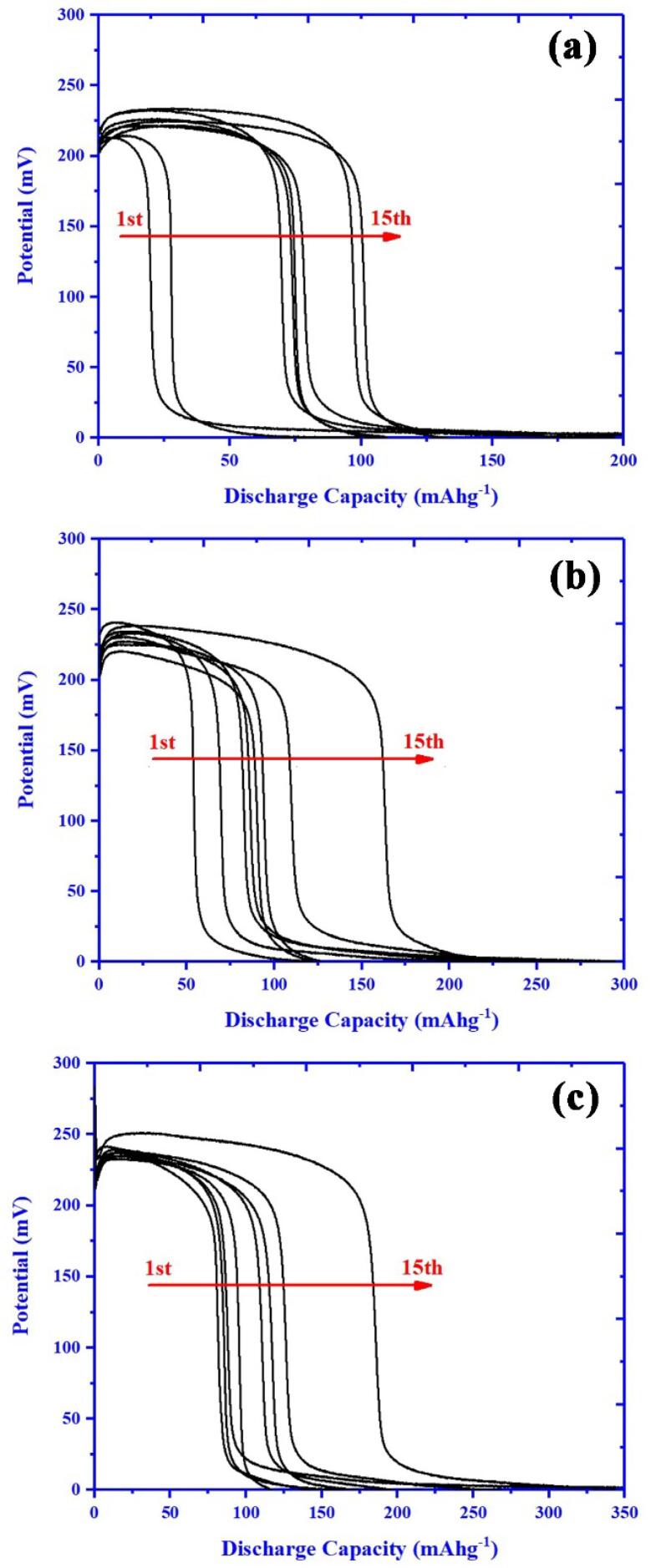
Discharge profiles at constant current of 1 mA for resultant samples (a) BaMoO_4_/ZnO, (b) BaMoO_4_/ZnO-GO and (c) BaMoO_4_/ZnO-GQDs.

The hydrogen storage capacity of BaMoO_4_/ZnO and representative nanocomposites were investigated using chronopotentiometry test. The discharge profiles for resultant electrode materials of BaMoO_4_/ZnO, BaMoO_4_/ZnO-GO and BaMoO_4_/ZnO-GQDs show in Fig. 8 a-c respectively. The capacity in first cycle for BaMoO_4_/ZnO, BaMoO_4_/ZnO-GO and BaMoO_4_/ZnO-GQDs are about 60, 106 and 132 mAhg^−1^ respectively. The discharge capacity after 15 cycles for BaMoO_4_/ZnO calculated about 129 mAhg^−1^. The discharge capacity modified in the presence of GQDs and GO and enhanced to 284 and 213 mAhg^−1^ after 15 cycles correspondingly. The previous active materials which applied for hydrogen storage application listed in Table. 4. By comparison of these materials with BaMoO_4_/ZnO based materials (this work), effect of BaMoO_4_/ZnO, BaMoO_4_/ZnO-GO and BaMoO_4_/ZnO-GQD materials as potential hydrogen storage materials confirm.

**Table 4:** Comparison of different active materials applied in electrochemical hydrogen storage systems with the results of this work.

No.	Material	Electrolyte	Capacity (mAhg^−1^)	Ref.
1	ZnMn_2_O_4_	KOH (2 M)	160	[94]
2	Ce_2_W_2_O_9_/CoWO_4_	KOH (2 M)	115	[17]
3	NiMnO_3_-RGO NiMnO_3_	KOH (3 M)	91 47.7	[95]
4	CoMn_2_O_4_	KOH (1 M)	134	[96]
5	LaCrO_3_	KOH (7 M)	107	[97]
6	LaFeO_3_	KOH (7 M)	80	[98]
7	Li_2_Ni(WO_4_)_2_ Li_2_Co(WO_4_)_2_ Li_2_Cu(WO_4_)_2_	H_2_SO_4_ (0.5 M)	121 98 84	[99]
8	BaMoO_4_/ZnO BaMoO_4_/ZnO-GO BaMoO_4_/ZnO-GQD	KOH (2 M)	129 213 284	This work

ZnO with enriching oxygen vacancies appears to be a capable substitute surface alteration substantial for educating capability owing to its high electronic conductivity, facile construction and low cost. Thus, presence of ZnO in the BaMoO_4_/ZnO texture with high electronic conductive ZnO would be reasonably valuable for storage capability [81].

The mechanism for hydrogen storage in the alkaline electrolyte of potassium hydroxide mentioned in bellow:

According to the conversion of water to hydroxyl ions with electron transfer, the hydrogen was produced which mentioned as Volmer reaction (Eq. (14)). then, this formed hydrogen adsorbed on the providing working electrode in the form of XH_ads_ (Eq. (15)). Based on Eqs. (16) and (17) which known as Tafel and Heyrovsky, the H_2_ was provided during recombination process. So, the discharge occurred as Eq. (18).

(14) $$H_{2}O+e^{-}↔H+OH$$

(15) $$X+H\to XH_{\mathrm{ads}}$$

(16) $$XH_{\mathrm{ads}}+H_{2}O+e^{-}\to H_{2}+OH^{-}+X$$

(17) $$2H↔H_{2}$$

(18) $$XH_{\mathrm{ads}}+XH_{\mathrm{ads}}\to H_{2}+2X$$

The redox process can be happened as Eq. (19) by reduction of Mo^6+^ to Mo^5+^. Then, the charge balance was occurred by hydrogen adsorption in the BaMoO_4_. The compound approved the organization of hydroxyl anions with a reduction of Mo^6+^to Mo^5+^ over the generation of bonds between molybdenum and hydrogen [17].

(19) BaMo^(VI)^O_4_ + ·H_2_O + x e**^-^** ↔ BaMo^(VI)^_1-x_Mo^(V)^_x_O_4_ -H^(I)^_x_ + x OH**^–^**

(20) (BaMoO_4_)-H_ads_**+ (**BaMoO_4_)-H_ads_ **↔** 2 BaMoO_4_ **+** H_2_

The hydrogen storage capacity for BaMoO_4_/ZnO-GQDs and BaMoO_4_/ZnO-GO modified against BaMoO_4_/ZnO due to the rapid electron transfer in the carbon-based materials. The GQD with rich edges areas and high conductivity leads to capacity development in the interaction with BaMoO_4_/ZnO constructions. The GQDs can be planned the electronic states owed to location of great amount of its atoms on the surface. These surroundings can act as electron or hole traps which concentrating at the surface. The surface properties of quantum dot are an exceptionally dynamic part, coordinated by chemical types which can have significant effects on the of the quantum dots performances. These properties can lead to manufactures of confined electronic states or extremely reactive sites, which are motivated to chemical and redox events [82].

Also, conductive system of GO produced the electron transportation ways. Next, the electrical conductivity of the active materials is upgraded and the hydrogen diffusion between crystal lattices and electrolyte is eased. These outcomes show that capacity can be obviously upgraded by graphene oxide nanocomposites. [83]. These astonishing electrochemical assets of carbon-based nanocomposites make it an optimistic candidate for using in marketable electrochemical storage policies.

## 4 Conclusion

In summary, BaMoO_4_/ZnO nanostructures was sonochemically synthesized in the short possible time in the presence of tween 20 with scales-like morphology. Also, GQDs was synthesized through hydrothermal method using natural source of *Spiraea crenata* to compare with GO. The specific surface area for BaMoO_4_/ZnO-GQDs (11 m^2^/g) and BaMoO_4_/ZnO-GO (124 m^2^/g) nanocomposites increased by comparing to BaMoO_4_/ZnO (9.1 m^2^/g). The introduced nanocomposite of BaMoO_4_/ZnO-GQDs and BaMoO_4_/ZnO-GO were scrutinized as electrode materials for hydrogen energy storage. Presented nanocomposites were identified with different methods and tested using electrochemical routs of CV and CP. The results show capacity of 129, 213 and 284 mAhg^−1^ after 15 cycles in the 2 M KOH electrolyte at 1 mA current for BaMoO_4_/ZnO, BaMoO_4_/ZnO-GO and BaMoO_4_/ZnO-GQDs which confirm the effect of carbon-based additives on the developing electrochemical behaviors of nanostructured BaMoO_4_/ZnO.

## CRediT authorship contribution statement

**Fatemeh Karkeh-Abadi:** Software, Investigation, Methodology, Writing – original draft, Formal analysis. **Maryam Ghiyasiyan-Arani:** Project administration, Formal analysis, Data curation, Investigation, Software, Writing – review & editing, Writing – original draft. **Masoud Salavati-Niasari:** Formal analysis, Methodology, Writing – review & editing, Writing – original draft, Conceptualization, Supervision, Project administration, Investigation, Data curation, Validation, Resources, Visualization, Funding acquisition.

## Declaration of Competing Interest

The authors declare that they have no known competing financial interests or personal relationships that could have appeared to influence the work reported in this paper.

## Data Availability

The authors do not have permission to share data.
